# Critique of the concept of motivation and its implications for healthcare practices

**DOI:** 10.1186/s13010-019-0083-6

**Published:** 2019-10-22

**Authors:** Leonardo Augusto Negreiros Parente Capela Sampaio, José Ricardo de Carvalho Mesquita Ayres

**Affiliations:** 1 Department of Preventive Medicine, University of São Paulo Medical School, Av. Dr. Arnaldo, 455, 2° andar, São Paulo, Brazil; 2 Department and Institute of Psychiatry, University of São Paulo Medical School, São Paulo, Brazil

**Keywords:** Motivation, Stages of change, Phenomenology, Philosophy of medicine, Empirical psychology, Motivation, Étapes du changement, Phénoménologie, Philosophie de la médecine, Psychologie empirique

## Abstract

**Background:**

Motivation is a crucial and widespread theme within medicine. From clinical to surgical scenarios, acquiescence in taking a pill or coming to a consultation is imperative for medical treatment to thrive. The “decade of the brain” gave practitioners substantial neuroscientific data on human behavior, helped to explain why people do what they do and created the concept of “motivated brain”. Findings from empirical psychology stratified motivation into stages of change, which became more complex over the decades. This research seeks to improve the understanding of how people make decisions about their health, and how to better understand strategies and techniques to help them resolve ambivalence in an effective goal-oriented way.

**Methods:**

We establish a dialogue with Ricoeur’s phenomenology of the will in order to understand the meaning of these scientific findings. Starting from Husserlian phenomenology, Paul Ricoeur developed his thoughts away from transcendental idealism, through emancipating the intentional structures of the will from the realm of perception.

**Results:**

Through introducing the concepts of the voluntary and the involuntary, Ricoeur deviated from Cartesian dualism, which renders the body as an object body, a target of natural vicissitudes. The new dualism of the voluntary and the involuntary is dealt with by reference to what Ricoeur called the central mystery of incarnate existence, which considers man “double in *humanity*, simple in *vitality*”. This duality makes it possible to consider the brain to be the natural organ of behavior in the human body, and to use empirical psychology as a path to escape from shallow subjectivations of concepts.

**Conclusions:**

Paul Ricoeur’s simplicity (or unity) of existence provides an invitation for medicine to rethink some of its philosophical assumptions, such that patients can be considered to be autonomous subjects with authorial life projects. Ricoeurian anthropology has a deep ethical impact on how medicine should use technology, which arises from empirical psychology findings. The usage of this new knowledge also needs to be thoroughly inspected, since it shifts the social role of medical science.

## Résumé

**Introduction:**

La motivation est. un thème crucial et répandu en médecine. Que. ce soit pour un scénario clinique ou chirurgical, l’acceptation de prendre une pilule ou de se rendre à une consultation est. essentielle au succès du traitement médical. La “décennie du cerveau” a fourni aux praticiens des données neuroscientifiques substantielles sur le comportement humain, a aidé à expliquer pourquoi les gens font ce qu’ils font et a créé le concept de “cerveau motivé”. Les résultats de la psychologie empirique ont stratifié la motivation en étapes de changement, qui sont devenues plus complexes au fil des décennies. Cette recherche vise à améliorer la compréhension de la façon dont les gens prennent des décisions concernant leur santé et comment mieux comprendre les stratégies et les techniques pour les aider à résoudre les problèmes d’ambivalence de manière efficace et ciblée.

**Méthodes:**

Nous établissons un dialogue avec la phénoménologie de la volonté de Ricoeur afin de comprendre le sens de ces découvertes scientifiques. À partir de la phénoménologie husserlienne, Paul Ricoeur a développé sa pensée en s’éloignant de l’idéalisme transcendantal en émancipant les structures intentionnelles de la volonté du domaine de la perception. Résultats: En introduisant les concepts de volontaire et d’involontaire, Ricoeur s’est. écarté du dualisme cartésien, qui fait du corps un corps d’objet, cible de vicissitudes naturelles. Le nouveau dualisme entre volontaire et involontaire est. traité par référence à ce que Ricoeur a appelé le mystère central de l’existence incarnée, qui considère l’homme “double dans l’*humanité*, simple dans la *vitalité*”. Cette dualité permet de considérer le cerveau comme l’organe naturel du comportement dans le corps humain et d’utiliser la psychologie empirique comme moyen d’échapper aux subjectivations superficielles des concepts.

**Conclusion:**

La simplicité (ou unité) d’existence invite la médecine à repenser certaines de ses hypothèses philosophiques, de telle sorte que les patients puissent être considérés comme des sujets autonomes avec des projets de vie d’auteur. L’anthropologie ricourienne a un impact éthique profond sur la manière dont la médecine devrait utiliser la technologie, ce qui découle de résultats de psychologie empirique. L’utilisation de ces nouvelles connaissances doit également faire l’objet d’une inspection minutieuse, car elle modifie le rôle social de la science médicale.

## Background

### First thoughts on motivation and medicine

Motivation is a crucial and widespread theme within medicine. The daily act of prescribing a medicine is necessarily followed by patients’ reflection on whether they feel like taking it when they gets home, or not. This is even more dramatic in relation to surgical procedures, since consent alone is not enough. Healthcare professionals need to “keep patient motivation up” through medical examinations and oscillations of will that may lead to dropout. Keeping a person as an inpatient for days or months is a constant exercise of reinforcing relational bonds and strengthening motivation to engage in treatment.

What is motivation, though? What does this concept refer to? A motivated person is an agent of action, an action towards self-healthcare or some other objective. But how is this agent perceived? Is the human being conditioned by environmental determinants of behavior, performing actions that can be statistically predicted, and shifted, through specific techniques? Can there be more to this definition? What consequences to healthcare practices will follow if human beings are considered as something other than exclusively the object of natural sciences? What impact will there be, on the one hand, on the scientific agenda for investigating the motivated brain; and on the other hand, on the (soft) technology produced within this framework?

Considering the classic Leavell and Clark model [1] for primary, secondary and tertiary prevention, all levels require the patients to be motivated to allow interventions relating to the natural history of diseases. Fighting sedentary lifestyle through health education, vaccinating children or referring cancer patients to psychotherapy requires interventions that all involve negotiation between doctor and patient or family about a diagnosis, and ways to treat it.

Since healthcare is “all about motivation”, it is crucial for medicine to (re) think the philosophical aspects of this concept in order to (re) arrange the framework that is used to define it, analyze its critical background and produce practices that derive from it. The role that empirical research (such as brain imaging, but also neuropsychological or endophenotypical findings) plays in explaining the concept of motivation needs to be taken into account, along with its consequences regarding how human beings are cared for through medicine and the care process itself. Therefore, in this essay, scientific, empirical or objective data are considered not as mirrors of a given reality, but rather, as human constructs that entail production of meaning for a perceived phenomenon [2].

When referring to empirical research findings, it is not of our interest to address the results themselves, to discuss the methods or statistical treatment applied to the data, but to seek understanding [2] on the philosoplical assumptions upon which the scientific community delineates its objects (and, therefore, produce such data), and which consequences are draw from the results. New researches and new findings bring to light not only crude data, but series of interpretations made by scientists within scientific narratives, that claim different discernments on old topics, and perhaps new ones.

We will not consider “motivation” only as a concept to be described by the pronouncements derived from the natural sciences, their research findings and epistemological deliberations. It is of our interest to understand the potential results of these new narratives on the comprehension of what motivation is, especially when there is technology produced by this movement. Health technology may reflect the assumption that a certain patient is depleted of motivation, therefore requiring top-down interventions to restore proper “motivational tone”, as if we were describing a blood transfusion; or may consider this same patient a “being-in-the-world” [3] that learned to get acquainted with his or her own body in a way that, even though there is a voluntary part of the movement (taking a medication, for example), there may be also layers of motivation not completely explicit at that point (What is the meaning of taking this medication? How does it feel to be sick? Who am I in the relationship with this doctor?), even to the patient.

What assumptions do scientists take into account when they compose the narratives that delineate the objects they use to conceive their researches and produce empirical findings? What does it mean for the scientific community to obtain new neuropsychological or neuroimaging data? How does this new discovery fit (or change) the conception of the object that was there before the research? How do undeclared assumptions about the conception of human being lying underneath scientific narrative relate to health technologies derived from objectively produced empirical data?

### The brain takes the lead

“My crown is in my heart, not on my head;

Not decked with diamonds and Indian stones,

Nor to be seen: my crown is called content:

A crown it is that seldom kings enjoy”[William Shakespeare, Henry VI, part 3, Act 3, Scene 1]

Henry’s crown may have been in his heart, as are love, emotions and passions in the world of poetry. Nonetheless, if a real king nowadays says something inappropriate or behaves in an awkward manner, this will probably lead his physician to recommend that he should undergo brain magnetic resonance imaging. The “decade of the brain”, as the 1990s were designated, was an important stage in brain imaging that led to production of incredible neuroscientific data [4]. These findings could be interpreted as a solid basis for future research, or as (quasi-)ready endophenotypic models that would help neuroscientists and doctors to shed light on the reasons why people do what they do.

The idea of a “motivated brain” that affective and social neuroscience gauges through neuroimaging was made possible by techniques that enabled visualization of the live cerebral networks involved in cerebral processing of emotional, motivational and social stimuli [5]. Moreover, interest in using the philosophy of medicine and psychiatry to understand and analyze the fundamental concepts of medical practice has grown over recent years. Nevertheless, contributions towards studying the role of neuroscientific data in the conception of man in medicine remain welcome, especially with regard to planning healthcare interventions that are nonviolent and respect patient involvement through empowerment and self-management [6].

What does it mean to say that Hispanic males have more motivation to stop substance abuse than non-Hispanic males, since they score higher on desire for help scales [7]? How can we interpret the data that brain response to sexual stimuli compared to neutral stimuli is the activation of right superior parietal lobule and left inferior parietal lobule [8]? These findings have great impact on increasing our understanding of the human brain and how it establishes relations to the environment. On the other hand, no automatic philosophical reflection is produced within the research framework.

The possibility of reporting objectively what has been seen is in different compass between the “results” and “discussion” sections in a paper. While one could attempt to present statistical data as what has been crudely drawn from scales and measurements (even though statistical treatment of the data is far from an absolutely linear and logical path), this is surely harder in the discussion, where the scientist must promote dialogue with the narratives that are already in the literature. Objectively produced data will now be interpreted as “making sense” or not, as evidences that add up to others or refute them. The scientist will choose among different ways to perceive the concept, the various traditions that produced considerations on the topic.

Statistical analyses may show unequivocally that Hispanic males are more motivated to treatment on a specific domain, but how the scientist will approach the concepts of race and ethnicity, the relevance given to the concern not to make it a stereotyped judgment, or how this finding relate to the qualitative experience of Hispanic males in substance abuse contexts; that is not implicit in the produced data.

How can all of the astonishing findings that come from neurosciences be interpreted? Do data found through brain research techniques point towards one sole path? Does this path necessarily unfold as the aftermath from a situation in which a human being who seeks care for a health problem is only the owner of a body that is to be unveiled by the natural sciences? Which philosophical assumptions lie beneath the conclusions that are drawn from neuroscientific data?

## Methods

In this study, we are not interested specifically in the findings that come from neuroscience, but in how they impact our understanding of what the object of medical science is. If the way medicine perceives the human being reflects the way in which doctors see and treat their patients, dissertations on the features of human motivation and human will might enlighten the path towards how, ultimately, we understand what medicine epistemologically and ontologically is.

In order to foment reflections on this topic, this research traces back the concept of motivation to two points in history at which its assumptions were revisited. One was the time when the notion of behavioral stages of change emerged within empirical psychology in the United States, in the late 1970s. This movement influenced what would come to be evidence-based psychotherapy, which became a hegemonic approach. The other was the time, just after the Second World War, when Paul Ricoeur (1913–2005) produced his doctoral thesis *Philosophie de la volonté* (Philosophy of the will), of which the first part, *The voluntary and the involuntary*, was published in 1950. Ricoeur’s thinking influenced the next decades of studies on the philosophy of action and phenomenology.

Ricoeur’s later reflections led him to examining different forms of extended discourse, such as metaphors. While logical propositions would say that something “is” or “is not”, metaphorical discourse had the power to say that something “is” and “is not”. According to Ricoeur, live metaphors do not produce mere decorative or rhetorical effect, but a redescription of reality. This drew the author’s attention to how people say things. “Who said that?”, “Who did that?”, and ultimately “Who is that?”, leading him to the concept of narrative identity and the turn to selfhood. In this paper, we have the Ricoeur who was concerned in forging the phenomenological foundations of this project, in his pre-hermeneutical philosophical anthropology. Our focus will be in understanding what is the conception of the human being to Ricoeur in this moment of his oeuvre, and how it contrasts with other narratives, such as the one produced by the empirical psychology scientific community in the late 1970s.

Enlighted by Gadamer’s philosophical hermeneutics [2], our aim here was to improve understanding in relation to two ways of perceiving the concept of motivation, which refer to two different philosophical backgrounds. We did not aim to judge the validity or reliability of these constructs, or whether they do or do not reflect the truth of a given fact distant from uncertainty; nor did we aim to decompose the units of these discourses, to analyze whether their statements are adequate for actually producing knowledge.

Rather, our objective was to promote a “fusion of horizons” (*Horizontverschmelzung*) [2] between each of these two perspectives and our own hermeneutic situation regarding the understanding of the concept of motivation, and the implications of this concept for the philosophical notion of the human being within medicine, and consequent healthcare practices.

## Results

### Stages of change and motivation

Psychology is a field that has been a matter of dispute since its very definition [9]. The basic aspects of a science are defined when its object is established and methods for studying this object are agreed within the scientific community. When Wilhelm Wundt created the first laboratory of experimental psychology [10], his conception of what this science should investigate was very different from what Sigmund Freud considered the object of psychoanalysis to be, years later [11]. In 1959, Harper identified 36 distinct systems of psychotherapy; in 1976, Parloff described 130 therapies; and in 2011, Pearsall estimated that there were over 500 [12].

In 1979, James O. Prochaska published the first edition of *Systems of psychotherapy: a transtheoretical analysis* [13], with the aim of achieving a “more integrative model of change”. The project compared these particular systems, discussed their concepts and filtered commonalities among them. Prochaska came up with the idea that behavioral change could be boosted through therapeutic interventions that could be classified on a spectrum from awareness or insight therapies, to action or behavioral therapies [12]. As a core concept for scientific psychology, it would then be possible to dissect and categorize change processes.

Prochaska started to develop this work in 1982, based on the temporal idea of the four stages of change in modifying health-related behavior: contemplating change, deciding to change, short-term change and long-term change. In this first study, subjects were asked to relate their experiences of the change process to three periods of change: decision to change, active change and maintenance [14].

### Changes to the stages of change

The idea of integrating techniques from different psychological theories inspired research on the stages of change. Starting with three stages in the article of 1982, this number was reviewed in 1983 [15]. Precontemplation, contemplation, action and maintenance were the new steps towards behavioral change that were described within Prochaska’s empirical psychology. New revisions of the transtheoretical model were put forward over subsequent years, thereby increasing the number of stages and deepening the comprehension of how to help people to move on to the next stage. Table 1 shows how the understanding of stages of change shifted over the years, and the authors who described these stages.

**Table 1:** Evolution of the stages of change in the 1980s and 1990s

Year	Stages of change	Authors
1982 [14]	Decision to quit Active change Maintenance	DiClemente, Prochaska
1982 [16]	Contemplation Determination Action Maintenance	Prochaska, DiClemente
1983 [17]	Precontemplation Contemplation Action Maintenance	McConnaughy, Prochaska, Velicer
1983 [15]	Precontemplation Contemplation Action Maintenance	Prochaska, DiClemente
1991 [18]	Precontemplation Contemplation Preparation Action Maintenance	DiClemente, Prochaska, Fairgurst, Velicer, Velasquez, Rossi
1992 [19]	Precontemplation Contemplation Preparation Action Maintenance Termination	Prochaska, DiClemente, Norcross
1994 [20]	Precontemplation Contemplation Preparation Action Maintenance	Prochaska, Velicer, Rossi, Goldstein, Marcus, Rakowski, Fiore, Harlow, Redding, Rosenbloom, Rossi
1997 [21]	Precontemplation Contemplation Preparation Action Maintenance Termination	Prochaska, Velicer

Based upon these empirical findings, Prochaska and other groups of researchers became interested in developing techniques to treat patients with specific health conditions, regarding their motivational deficits. Prochaska then gathered a set of techniques derived from the different branches of psychology and unified under one name, transtheoretical analysis [13]. The initial aim of this therapy was to help patients with dependency on or abuse of specific substances, such as nicotine. When better comprehension of motivational processes arose, this scope widened to broader behaviors, including radon gas exposure, exercise acquisition and physicians’ practices [20].

The next step towards improving healthcare techniques was to organize these principles in a structured way, in order to conduct counseling effectively. Motivational interview-based health coaching [22] would then be the strategy to help resolve ambivalence, through interventions that would be specific to the stage of change that the patient was in. Motivation as a natural concept to empirical psychology could now individualize counseling programs and boost behavior change through the development of stage-specific techniques.

However, it becomes crucial do ask: Could “motivation” have a different meaning? A point of view connected to another approach towards the human being? Could thoughts from a different philosophical background acknowledge the findings of empirical psychology and still enlighten the issue through this new perspective? The phenomenology of the will seems to bring us a fruitful parallel path, which might be important to bring better understanding on this topic.

## Discussion

### Paul Ricoeur and the philosophy of will

Paul Ricoeur dedicated a large part of his research to the *phenomenological hermeneutics of the self*, a philosophical anthropology that was perceived through an interpretative description [23]. Initially, in Ricoeur’s oeuvre, he turned to Husserl’s book *Ideas* [24], in which the latter author sought a path towards pure phenomenology. He was born in 1913, and had a life marked by tragic losses and important biographical turns from the outset. His mother died right after his delivery, and his father died when he was 2 years old, although the body would only be found 17 years later in a war camp. In times of scarcely developed communal leisure and entertainment media, Ricoeur’s life between the ages of 11 and 17 years centered mainly on his home and school. By the age of 20, he was already a teacher at the lyceum, and by 35, philosophy professor at the university [25].

When World War II started in 1939, Ricoeur joined the military. However, he became a prisoner of war the next year, and was held in a Nazi camp in Pomerania from 1940 to 1945, when the war ended and Canadian forces released the prisoners in that camp. During his years of captivity, he maintained his penchant toward German culture and deepened his studies on Jaspers and Husserl. Ricoeur even translated Husserl’s *Ideas pertaining to a pure phenomenology*^1^ into French, on the edges of the book pages [25].

### Objective data and Husserlian eidos

Ricoeur worked on his ideas of the phenomenology of the will during his captivity, although his first drafts on this issue had started in 1933 [25]. While reading and translating the *Ideas*, he realized that Husserl’s philosophy drew attention to perception as the path towards finding pure phenomenology, and this framework was an invitation to question this privilege. The structure of transcendental consciousness described by Husserl could be elucidated through the original lived experience, and the elements within it. The relationship between material impression and intentional content was crucial, according to Husserl [26], since these characterized different layers of the lived experience. The material layer was composed of data on sensations, which Husserl named hyletic data, and had no meaning by itself. Intentional acts or intentional experiences, on the other hand, were units of consciousness that were presented at the moment when a question was put forward, which would give meaning to a specific arrangement of “objective” data.

In Husserl’s view, there were non-intentional experiences such as pain, but intentional content would only appear when *hylé* (matter) summoned up the intentional act of fabricating meaning. The projection of hyletic data to consciousness, which was covered and transformed by intentionality would thus form lived experiences that aimed to provide data of the senses, which Husserl called *noema*. On the other hand, intentionality had another aim towards itself, the lived experience of the intentional act, called *noesis*. When *noesis* used *noema* to access *hylé*, the phenomenological description of a given act would need to be given exactly as it was experienced by the subject. Therefore, the content of the intentional act would need to be “bracketed”, focusing on the aspects of one’s intentional experiences that remained unrelated to the existence of a represented object. This reduction to experience itself, the phenomenological *epoché*, would signify the essence of the phenomenon, i.e. its *eidos*.

### The turn from perception to the will as the keystone of lived experience

Husserl did mention that lived experiences in the affective and volitional spheres fell within the same correlational approach in terms of *noesis* and *noema*, as lived experiences of perception. However, this perspective still put lived experiences of the will in a position that was subordinate to perception. Husserl did not see the will as another gateway for accessing transcendental consciousness, but as a kind of indirect spawn that would have to start with *hylé.* On top of a perceptive core, intentionality would add progressive layers of meaning, which would then build the *noema* of the will.

Consciousness is also differently perceived by Husserl and Ricoeur. While Husserl sees it as fundamentally rational, Ricoeur highlights its practical feature.^2^ Ricoeur advocates in favor of this particular *noema*, “the willed”.^3^ This is not the will as a psychological ability or a natural object, but the lived experience of willing to do something or acting [28].

Ricoeur freed the phenomenology of the will from the tutoring of the phenomenology of perception and granted it the status of autonomous objectivity. Human action would not need to start its ontological loop from the “facts of consciousness” anymore; it could now be a gateway on its own. Another consequence of this line of thought was in relation to the nature of being itself. While Husserl aimed to edify phenomenology over the unity of the world, Ricoeur would now risk pluralizing its ontology [28]. “The willed” could now be the wellspring from which the river of (practical) consciousness flowed, thus enabling description of the intentional structures of this consciousness, which belonged to an autonomous subject. Ricoeur described his study *The Voluntary and the Involuntary* as “an eidetic of the voluntary and the involuntary, provided that we remain constantly on guard against any Platonizing interpretation of essences” [28].

### A “free will”

When Paul Ricoeur allowed the phenomenology of the will to enlighten the path towards pure description of human actions without subordination to perception, it became necessary to study the fundamental structures of the voluntary and the involuntary. He described a triadic interpretation of the act of the will, formed by three attitudes that together mean “I will”: “I decide”, “I move my body” and “I consent”. The “I decide” portion referred to the thing that I decide, my authorial project, followed by a voluntary movement, an action. Ricoeur consider there was still a residuum, i.e. that there was more to the willed than deciding and doing: “The will does not resolve into an empty project and its practical execution in action”. There would also be acquiescence to the need that motivated the action, i.e. the one that drove the decision. The “because” of motivation would therefore lead to a detour into the realm of the involuntary, of consenting to need, pleasure, pain, etc., and to “the ‘I’ of the Cogito”^4^ [30].

In order to understand the relationship between these intentional structures, Ricoeur acknowledged that they were incarnated in a body. However, the body that one would experience as “mine or yours” would differ from the body as an object among the objects of science, i.e. the object body. Since these are the same body, it would now be necessary to correlate them.

“Any moment of the Cogito can serve as an indication of a moment of the object body – movement, secretion, etc. – and each moment of the object body is an indication of a moment of the body belonging to a subject, whether of its overall affectivity or of some particular function. ( … ) Such analysis of symptoms, which we are here using with respect to the Cogito, is used by a doctor in service of empirical knowledge, an experience indicating a functioning or a functional disorder of the object body. But the two points of view are not cumulative; they are not even parallel. The use of the descriptive method shows that the lessons of biology or of empirical psychology are a *normal* path for discovering the subjective equivalent which is often quite ambiguous. In some cases it will appear almost impossible to discover the subjective indication, in the language of the Cogito, of a function or an occurrence which is well known in biology or in empirical psychology” [30].

Far from purposing a psycho-physiological parallelism, Ricoeur was investigating how these intentional structures related to the ontology of human action. The concept of “motivation” reflected the connections between the voluntary and the involuntary that would drive someone to a specific project. The function of the “will” that is described by empirical psychology would therefore be a purpose of science that dwelled at the object body. The functional stratification of the will might be the usual path towards unraveling its subjective dyad, but there is more.

“On the one hand understanding of the structures of the subject constantly refers to empirical and scientific knowledge which serves as a symptom of such intentional structures, while on the other hand fundamental articulations of these structures reveal the unity of man only by reference to a central mystery of incarnate existence” [30].

What is the decision-making process of this body that is animated by motivation? How can projects for people’s own lives be built upon an involuntary background? These questions need to be answered through a lens that acknowledges the issue of a dualistic explanation for this subject matter. Describing the foundations of intentional structures that reveal the unity of man needs to be discussed through subdividing human action into voluntary and involuntary.

### The trap of the human brain

Paul Ricoeur referred to the Cartesian duality of *res cogitans* and *res extensa* in order to understand the relationship between the voluntary and the involuntary. While Descartes highlighted the certitude of the submissiveness of the body that can be observed when people’s legs move after they have willed a walking action, Ricoeur drew attention to the reflection that the bond between these two *res* was “polemic and dramatic” [30]. According to Ricoeur, this submissiveness was not granted, but conquered. The idea of moving one’s body right after making a decision to do so was not a birthright but an ability that can be developed in a crude instrument. Individuals need to become acquainted with the totality of their neuroanatomical and neurophysiological apparatus so that they can become adept at using it. “There are no voluntary acts that have not firstly been accomplished involuntarily” [30]. Here, Ricoeur establishes the involuntary as a background that make it possible for voluntary action to be identified, noticed and recognized.

Although the voluntary and the involuntary were considered here to be two reciprocal parts, since voluntary action could only be perceived through the involuntary background, another form of dualism was introduced. In order to understand how the different facets of action could assemble around the “ontological unit of thought and movement, apart from the duality of willing and the involuntary”, Ricoeur quoted Maine de Biran: “Homo simplex in vitalitate duplex in humanitate”. While *humanity* might incorporate this dramatic duality, one’s glimpse of life would have to penetrate deeper into the “very organic pact inscribed in the involuntary powers of movement”, the “simplicitas in vitalitate” (simplicity in vitality) [30].

In order to describe this fine alignment of human action in unity and duality, Ricoeur used Rainer Maria Rilke’s poem that depicts a horse-rider who climbs into the saddle. At the same time carried and guiding, the rider freely moves to wherever he or she wants. Although the rider’s freedom is mediated, it can only be perceived through a perspective that considers the horse. “Track and turning. Yet at a touch, understanding. New open spaces. And the two are one. But are they?”. This is the relationship that people have with their brains: a freedom that is “human, only human” [30]. The atmosphere of people’s actions, decisions and motives is surely their own body, their own brain. Nonetheless, could people be trapped by their own brains and thus either be prevented from taking action or be forced into it?

This is a tough question, that relates to the philosophical definition of what mind is. At this point, Ricoeur is not interested in this topic, he seeks to offer a different comprehension on how it can be possible for us to have brains that in a way work like machines (neurotransmitters, neuroaxis, personality patterns) and not be trapped by a closed system. At the same time that neuroscientific findings enlighten the recondite meanders of the human soul, the perspective on what is a human being can be easily driven to perceive it as an automaton tied by neural circuitry. Ricoeur acknowledges the relevance of scientific psychology research, especially when allied to deep philosophical meditation.

The role of neuroscientific findings and the idea of man within the philosophy of medicine thus need to be discussed. Regarding scientific research, exemplified by Prochaska’s studies on motivation, and philosophical reflection, as seen in Ricoeur’s attempt to give account for the problem of the voluntary and the involuntary; we should now focus our pondering on how people make choices for their own lives and their health issues. What is the role of neuroscientific data in the conception of the human being? What are the impacts of this perspective on medical practice? What limitations does a person’s body (or brain) impose on that person’s projects for his or her life?

## Conclusions

### Further thoughts

Neuroscientific findings of a brain that is being tested in a situation that requires subject motivation provides us with data that do not have any automatic philosophical consideration. Banner and Thornton [31] pointed out the importance of this reflection, highlighting how the “new philosophy of psychiatry” was already a flourishing philosophical field, with considerable research at least since the 1990s. Crude data can be used to justify a notion of the human being enclosed only as a natural object, the victim of material forces and, within healthcare, the target of interventions that aim to align the person’s body with a specific concept of health. The same data can be interpreted as a justification of individuals as beings that are guided by the intentional structure of their will, gravid with meanings. Without adequate philosophical care, counseling and other healthcare interventions may neglect individuals’ autonomy over their own bodies and projects for their own lives, violate these subjects and create a fissure in the old Hippocratic ethical principle of “do no harm”, recently reread as a practical guideline by the so called quaternary prevention [32].

On the other hand, there is also the risk of overestimating the voluntary part of human action, thus reducing the complex issue of decision-making, which makes reference to the uniqueness of people’s projects for their own lives, to a matter of quasi-quantitative willpower or moral fiber. The techniques produced through the technology of empirical psychology would be perceived as injections of ardor into a soul that is depleted of eagerness to become someone better (fit, non-smoker, sunscreen adherent, etc). The logic of diseases as excesses or reductions of humor can be seen within this rationale, but it needs to be borne in mind, as pointed out by Canguilhem, that “an organism’s norm of life is furnished by the organism itself, contained in its existence” [33].

There are important ethical implications to new technologies that come from the findings of neuroscience. Should mental health practitioners be assigned the mission of resolving patient ambivalence to treatment? Is treatment ambivalence something to be resolved, or is it part of what illness phenomenologically is, i.e. an existential fraction that, if eliminated, might amputate the whole experience of being ill? Kottow and Kottow [34] cited Viktor von Weizsäcker in highlighting how “In exploring only the organism, medicine is neglecting essential components of existence and disease”. How can doctors absorb leading-edge techniques produced by science and use them in an ethical manner? How can a philosophical and ethical debate be promoted within the medical community to ensure that technical knowledge is incorporated into practice with due care for patients’ autonomy to be able to trace out an authorial life project?

It is possible to stratify people’s motivations for engaging or not engaging in an action using the stages of change. Describing someone as being at the stage of contemplation for smoking cessation, at the stage of preparation for safer sex or at the stage of action for sunscreen use is useful, reliable and relevant for guiding the planning of counseling. Motivational interviewing is a directive (goal-oriented) client-centered counseling style for helping clients to resolve ambivalence about behavioral change [23] that has been shown to be effective in relation to a variety of problem behaviors, such as in relation to mammography screening, adolescent delinquency, weight control and quitting cocaine use [20].

What may pass unadvised are patients’ perspectives regarding what paths they want to follow to improve their health levels and those they do not wish to follow. Care is needed so as not to mistake this attitude for medical abandonment by the physician. The point here is not to give up being at the side of a person who is sick but does not know about this disease and its pathophysiological characteristics to the same extent that the doctor knows about it. On the contrary, it is an approach to healthcare that takes a bet in the direction of patient autonomy and empowerment. The attitude towards cancer patients who have been trying unsuccessfully to quit smoking without help for decades and say that they just do not care anymore should not be one of simply giving up on them. The issue is whether it is believed that these clients will benefit more from a doctor who performs aseptic technical interventions that aim to move them from being a “smoker” to being a “non-smoker”; or from another one who believes that these patients can achieve this and is beside them at the moment when the decision is made, so that they can start together to use the technical interventions.

Shapiro took the view that medical students’ behavior of distancing themselves from patients was a consequence of the modernist biomedical paradigm. In order to promote human interactions, this author advocated that the comprehensive primacy of this paradigm should be questioned, and noted the starting points that might be used in formulating an ethic of imperfection, as used by David Morris. Along with Paul Ricoeur, Shapiro wrote: “This moral framework would be anchored in acceptance of the limited control we exercise in life and the imperfectability of life itself. This viewpoint suggests that we must learn to accept as well as to resist bodily vulnerability” [35].

Ricoeur highlighted how scientific psychology is of great help as a diagnostic tool, although this approach can depict human beings through their mechanical features, recognizing only the object body. Phenomenology, on the other hand, may bring “human back to human”, aside from its pure biology. However, on its own, phenomenology may be superficial, with concepts that need to be enriched with empirical data, so as not to become simple subjectivations.

“This is why our method will be most receptive with respect to scientific psychology, even though it will make only diagnostic use of it. Description of the Cogito will frequently recover from empirical psychology the vestiges of a phenomenology which it discovers there in an objectified and in some way alienated form. But with equal frequency a phenomenological concept will be no more than a subjectivization of a concept far better known along an empirical path” [30].

Enlightened by Ricoeur’s philosophical anthropology and Gadamer’s philosophical hermeneutics, it is also possible to establish a productive dialogue here with the Heideggerian ontological concept of Care (Sorge, in the original [3]) and to sustain that considering the dialectical relationship between the voluntary and the involuntary within bodily experience goes far beyond just “doing no harm”. However, there is more than this within good clinical practice.

Heidegger’s concept of Care refers to the expression of the multiplicity of kinds of being-in-the-world, “indicated by the following examples: to have to do with something, to produce, order and take care of something, to use something, to give something up and let it get lost, to undertake, to accomplish, to find out, to ask about, to observe, to speak about, to determine” [3]. If patients are not only the owners of a body that is to be unveiled through the knowledge produced by the natural sciences, they can also be perceived as being the bearers of autonomy and the power to self-manage their own health issues. The concern should be not solely on doing no harm through overmedicalization, as proposed by quaternary prevention [36], but should allow medical practice to offer itself as a way for patients to build authorial projects for their own health in a collaborative manner with the doctors they choose.

## Footnotes

1 The first book of Husserl’s *Ideas pertaining to a pure phenomenology* is published as *Ideas: General Introduction to a Pure Phenomenology* [24].

2 The term “practical” here refers to the Aristotelian concept of *phronesis*, or practical wisdom. Finding and naming diseases in someone’s body is only a technical activity, which may consider the patient’s experience or neglect it. Medical practice also needs to consider the ethics of diagnosis and treatment. The physician should be *phronimos*, i.e. one who possesses practical wisdom, prudence or virtue ethics. “Diagnostic medicine is not a *phronetic* activity” [27].

3 A better translation of *“le voulu comme tel”* [28] would have to consider that *“le voulu”* actually means “it that is wanted”, “the lived experience of willing”, or simply “the willed”, which is the *noema* that interested Ricoeur.

4 Ricoeur used the linguistic distinction of *I* and *Me*, or *Je* and *Moi*, as first-person singular forms, to distinguish between an “idem-identity” and an “ipse-identity” of subjectivity. The first (*I*, *Je*) refers to the existence of the subject as an entity in the world and the second (*Me*, *Moi*), dialectically linked to the first, expresses the reflexive and relational aspect (a narrative identity) that characterizes the human subject as one who *inhabits* a world, who understands this world, thus giving a meaning to it and to oneself [29].
